# Supplementary material for: Early alterations of social brain networks in young children with autism
Source: eLife. 2018 Feb 27;7:e31670. doi: 10.7554/eLife.31670 (PMC5828667; doi:10.7554/eLife.31670)
Supplement: Transparent reporting form [file elife-31670-transrepform.pdf]

eLIFE
1st Floor
24 Hills Road
Cambridge CB2 1JP, UK
P 01223 855340
W elifesciences.org
T @elife

eLife's transparent reporting form

We encourage authors to provide detailed information *within their submission* to facilitate the interpretation and replication of experiments. Authors can upload supporting documentation to indicate the use of appropriate reporting guidelines for health-related research (see EQUATOR Network), life science research (see the BioSharing Information Resource), or the ARRIVE guidelines for reporting work involving animal research. Where applicable, authors should refer to any relevant reporting standards documents in this form.

If you have any questions, please consult our Journal Policies and/or contact us: editorial@elifesciences.org.

Sample-size estimation

- You should state whether an appropriate sample size was computed when the study was being designed
- You should state the statistical method of sample size computation and any required assumptions
- If no explicit power analysis was used, you should describe how you decided what sample (replicate) size (number) to use

Please outline where this information can be found within the submission (e.g., sections or figure legends), or explain why this information doesn't apply to your submission:

Description of the sample is provided in the Methods, participants subsection. We recruited 120 toddlers and preschoolers. Out of those, we did not manage to apply the EEG cap on the head of 23 toddlers and preschoolers with ASD and 7 of their typically developing peers. We managed to acquire EEG data on 90 participants. Out of those, we had to exclude 28 ASD and 26 TD participants because of too many movement-related artefacts, unrepairable noisy signal, lack of interest, or insufficient amounts of epochs available for subsequent analysis. This was to be expected given the extremely sensitive population at study. Although sample size had not specifically been estimated for our study, the final total number of participants included (N=36) is within a reasonable range when compared to other experiment in the field that include very young participants.

Replicates

- You should report how often each experiment was performed
- You should include a definition of biological versus technical replication
- The data obtained should be provided and sufficient information should be provided to indicate the number of independent biological and/or technical replicates
- If you encountered any outliers, you should describe how these were handled
- Criteria for exclusion/inclusion of data should be clearly stated
- High-throughput sequence data should be uploaded before submission, with a private link for reviewers provided (these are available from both GEO and ArrayExpress)

Please outline where this information can be found within the submission (e.g., sections or figure legends), or explain why this information doesn't apply to your submission:

eLife Sciences Publications, Ltd is a limited liability non-profit non-stock corporation incorporated in the State of Delaware, USA, with company number 5030732, and is registered in the UK with company number FC030576 and branch number BR015634 at the address 1st Floor, 24 Hills Road, Cambridge CB2 1JP | August 2014

The experiment does not involve replication. The methods section, participants subsection) clearly outlines our data exclusion criteria based on to many movement-related artefacts, unrepairable noisy signal, lack of interest, or insufficient amounts of epochs available for subsequent directed connectivity analysis.

Statistical reporting

- Statistical analysis methods should be described and justified
- Raw data should be presented in figures whenever informative to do so (typically when N per group is less than 10)
- For each experiment, you should identify the statistical tests used, exact values of N, definitions of center, methods of multiple test correction, and dispersion and precision measures (e.g., mean, median, SD, SEM, confidence intervals; and, for the major substantive results, a measure of effect size (e.g., Pearson's r, Cohen's d)
- Report exact p-values wherever possible alongside the summary statistics and 95% confidence intervals. These should be reported for all key questions and not only when the p-value is less than 0.05.

Please outline where this information can be found within the submission (e.g., sections or figure legends), or explain why this information doesn't apply to your submission:

All the statistical tests used are clearly identified within the manuscript in the Results, Summed outflow, Region-to-region directed functional connectivity, Correlations with ADOS-2, PEP-3, VABS-II scores and gaze Proximity Index subsections. Statistical tests used are also clearly described in the Methods, Participants and Directed Functional Connectivity using Granger causality subsections. All the results presented in the manuscript use stringent significance threshold and have been corrected for multiple testing using the Benjamini-Hochberg procedure (BH step-up procedure) to identify the important from the trivial effects tested. Effect sizes are also reported where relevant. Table 1, Characteristics of Study Participants includes the exact N, Mean, SD, T-values and Exact P-values.

(For large datasets, or papers with a very large number of statistical tests, you may upload a single table file with tests, Ns, etc., with reference to sections in the manuscript.)

Group allocation

- Indicate how samples were allocated into experimental groups (in the case of clinical studies, please specify allocation to treatment method); if randomization was used, please also state if restricted randomization was applied
- Indicate if masking was used during group allocation, data collection and/or data analysis

Please outline where this information can be found within the submission (e.g., sections or figure legends), or explain why this information doesn't apply to your submission:

This information does not apply to the current experiment as we compare the directed functional connectivity in toddlers and preschoolers with ASD compared to their TD peers. Thus no randomization nor masking was used.

Additional data files ("source data")

- We encourage you to upload relevant additional data files, such as numerical data that are represented as a graph in a figure, or as a summary table
- Where provided, these should be in the most useful format, and they can be uploaded as "Source data" files linked to a main figure or table

- Include model definition files including the full list of parameters used
- Include code used for data analysis (e.g., R, MatLab)
- Avoid stating that data files are "available upon request"

Please indicate the figures or tables for which source data files have been provided:

We will provide the Matlab codes used for the analysis as well as the cleaned EEG files of the 36 toddlers and preschoolers included in the present experiment.
